# Supplementary material for: Sub-nanoscale atom-by-atom crafting of skyrmion-defect interaction profiles
Source: Sci Rep. 2020 Sep 4;10:14655. doi: 10.1038/s41598-020-71232-2 (PMC7474088; doi:10.1038/s41598-020-71232-2)
Supplement: Supplementary file 1 — Supplementary information. [file 41598_2020_71232_MOESM1_ESM.pdf]

Supplementary Materials

Sub-nanoscale atom-by-atom crafting of skyrmion-defect interaction profiles

I Gede Arjana, Imara Lima Fernandes, Jonathan Chico, and Samir Lounis

Supplementary Table S1: Magnetic anisotropy energy (MAE) of the defects. The average value of MAE per impurity-site is given in meV where a negative value indicates a preferred in-plane orientation of the magnetic moment.

	fcc Cr	hcp Cr	fcc Fe	hcp Fe
adatom	-0.97	0.20	-0.78	-1.30
dimer-1	-0.71	-0.03	-0.03	-0.26
dimer-2	-0.85	0.25	-0.60	-1.22
dimer-3	-0.92	0.26	-0.73	-1.26
compact trimer-1	-0.50	0.16	-0.33	-0.13
compact trimer-2	-0.19	0.15	-0.09	-0.41
corner trimer	-0.62	0.00	0.00	-0.14
linear trimer	-0.56	0.07	-0.01	-0.27
linear tetramer	-0.54	0.14	0.00	-0.27

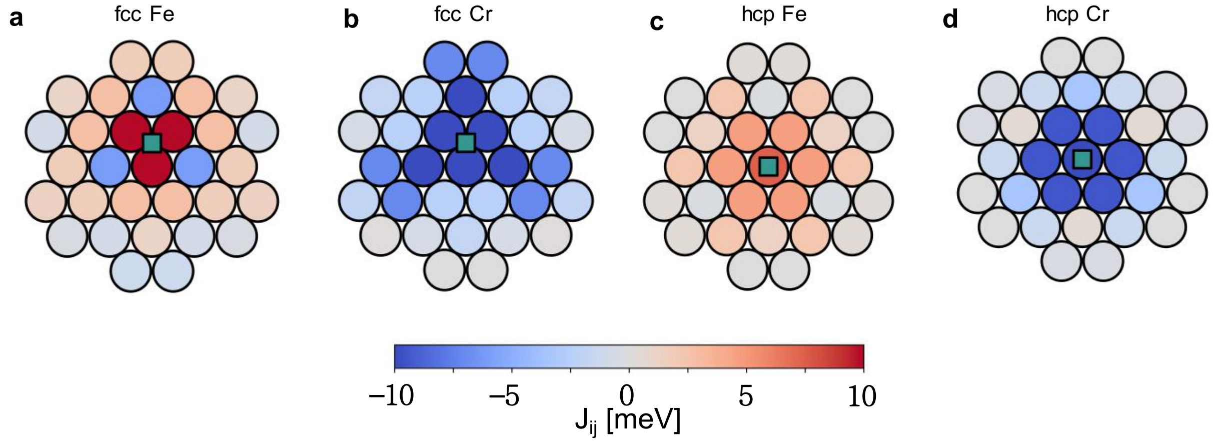

Supplementary Figure S1: Adatom-substrate magnetic exchange interaction. Long-range interactions are illustrated **a** fcc Fe, **b** fcc Cr, **c** hcp Fe and **d** hcp Cr adatoms. The spheres indicate the Fe atoms of the substrate while the green square shows the defect position.

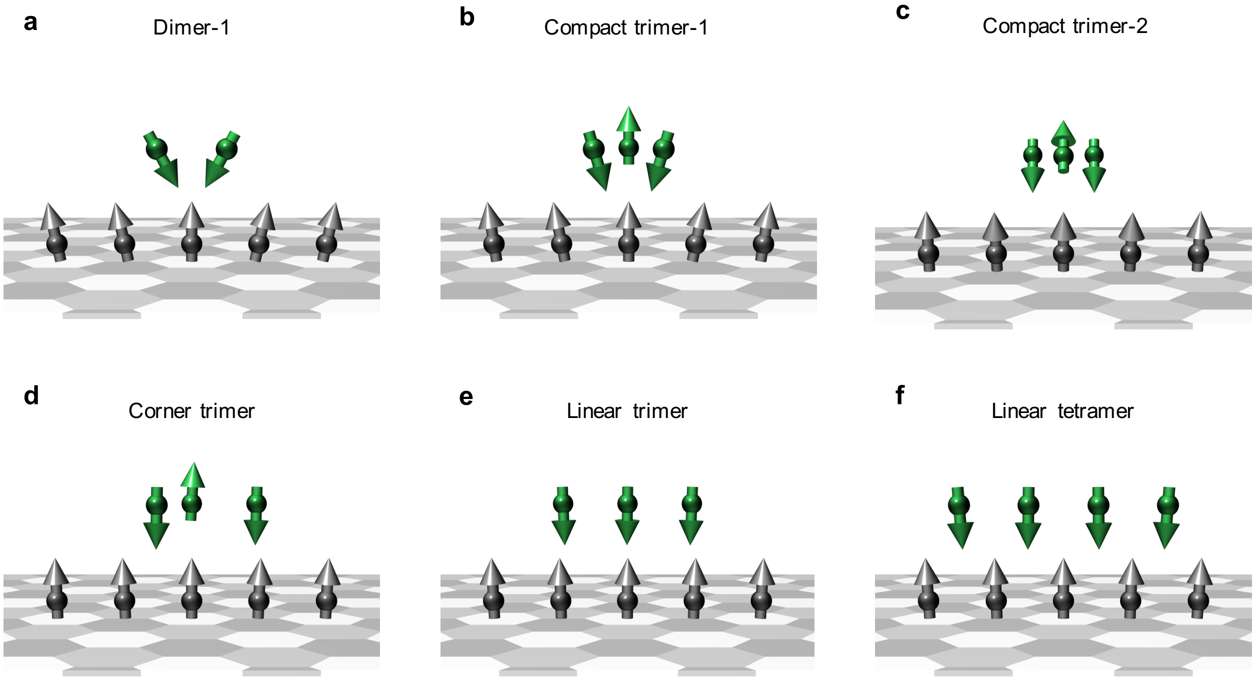

Supplementary Figure S2: Magnetic structure of fcc Cr nanostructures atop the ferromagnetic region. Non-collinear magnetic textures can occur owing to magnetic frustration between the antiferromagnetic intra-cluster and cluster-substrate magnetic interactions. While dimer-1, **a**, hosts a spin-flop state [1–4] (see Supplementary Note 3), the non-collinearity survives for compact trimer-1 (see Supplementary Note 4), **b**, but decreases strongly for compact trimer-2, **c**, and corner trimer, **d**, which host a ferrimagnetic state. Surprisingly, by opening the trimer to form the line, **e**, the three Cr moments align antiferromagnetically to the substrate magnetization thanks to the strong reduction of intra-cluster magnetic interactions. For similar reasons, the same collinear magnetic ground state is found for the linear tetramer, **f**.

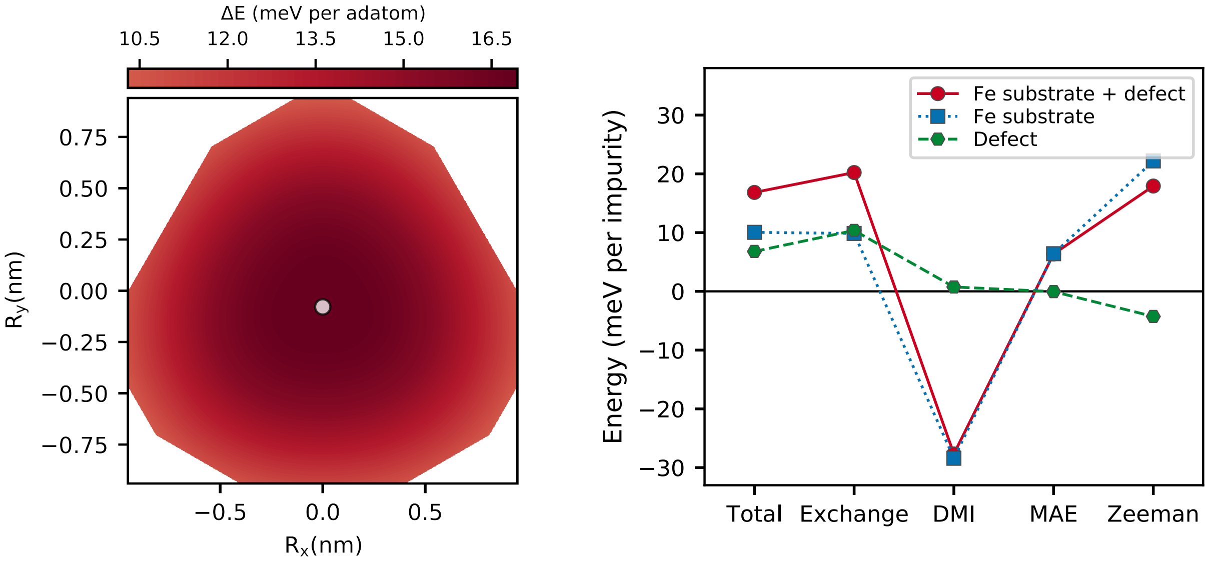

Supplementary Figure S3: Binding energy decomposition of a skyrmion interacting with an fcc Cr adatom deposited on PdFe/Ir(111). a Repulsive interaction pattern induced by the fcc Cr adatom. **b** The binding energy (red line) is decomposed into contributions from the Fe substrate (blue line) and from the defect (green line) when the skyrmion core is at position (0,0). Furthermore the binding energies are dissected into exchange, DMI, magnetic anisotropy and Zeeman contributions. As found analytically in Supplementary Note 1, the substrate's DMI energy counteracts that of the exchange (they have opposite signs). The DMI contribution is larger and helps pinning the skyrmion in this particular case. The Zeeman energy is, however, repulsive, which settles the interaction nature of the Cr adatom.

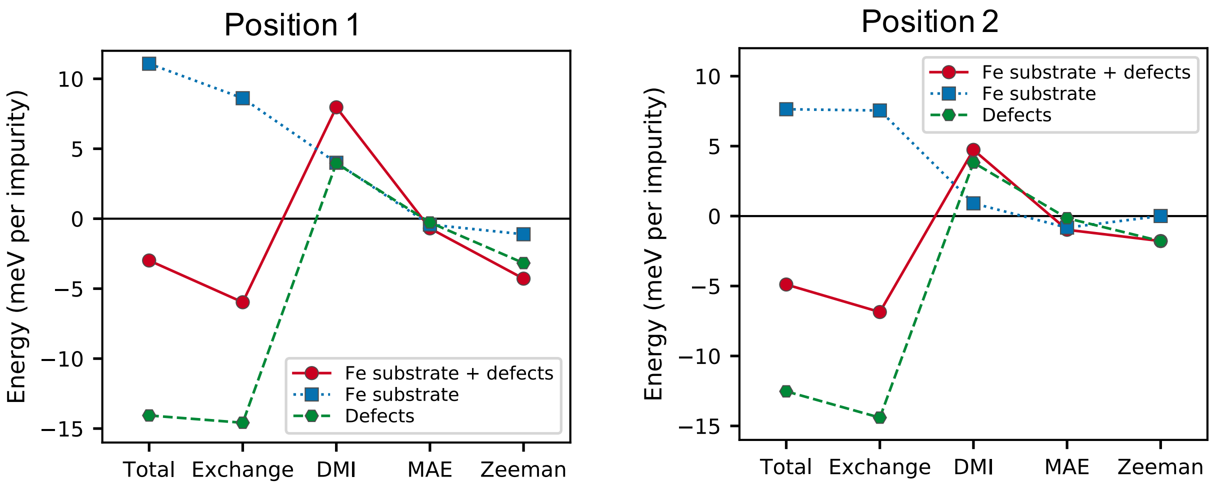

Supplementary Figure S4: Energy decomposition for Cr-dimer at the positions 1 and 2, at the vicinity of the skyrmion as discussed in the main text. At position 2, the substrate's DMI contribution is less repulsive than the one at position 1. This can probably be related to the degree of stiffness created at both positions. Overall it is the exchange contributions among the defects that leads to the pinning. In position 1 and 2, the angle between the Cr moments is larger than on the ferromagnetic region. Thus there is a strong gain in energy from the dimer side at the vicinity of skyrmion. The reason is that the effective substrate magnetization felt by the dimer is smaller in the skyrmionic region. The skyrmion, however, gets stiffen and that leads to a repulsive contribution to the exchange.

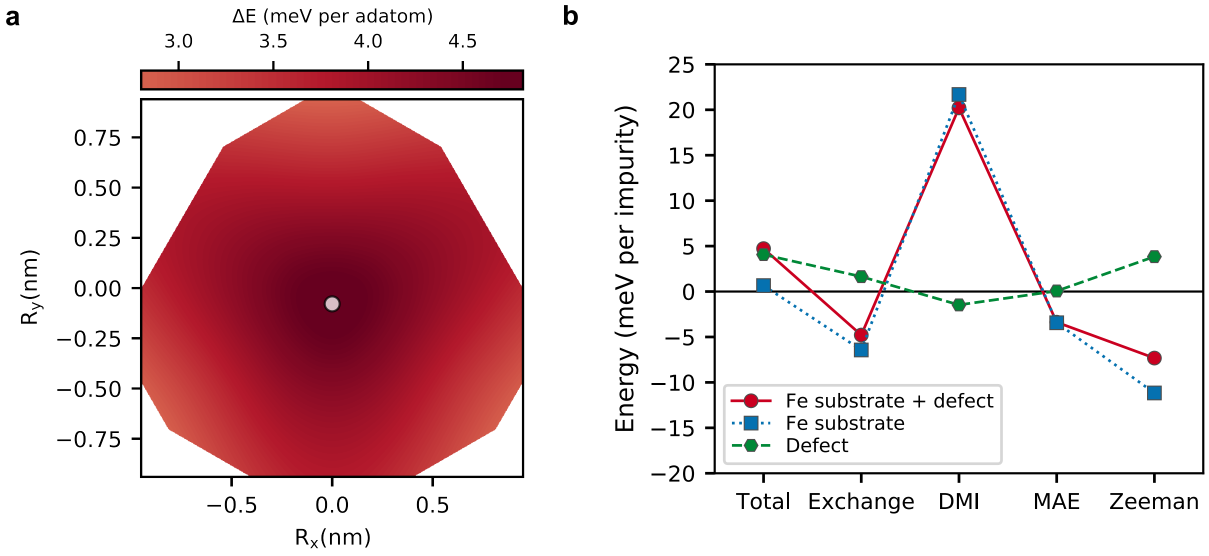

Supplementary Figure S5: Binding energy decomposition of a skyrmion interacting with by an fcc Fe adatom deposited on PdFe/Ir(111). a Repulsive interaction pattern induced the fcc Fe adatom. **b** The binding energy (red line) is decomposed into contributions from the Fe substrate (blue line) and from the defect (green line) when the skyrmion core is at position (0,0). Furthermore the binding energies are dissected into exchange, DMI, magnetic anisotropy and Zeeman contributions. In contrast to the Cr adatom, the DMI energy contribution is repulsive. The DMI contribution is strong enough to impose the repulsive behavior of the Fe adatom.

Supplementary Note 1 - Spatial dependence of the adatom-substrate magnetic interactions

The magnetic exchange interactions are not limited to the nearest neighboring atoms and oscillate as function of interatomic distances (see Supplementary Fig. S1). In the case of the fcc Fe adatom, for instance, the magnetic coupling between the adatom and the substrate's Fe atoms switches from ferromagnetic (FM) to antiferromagnetic (AFM) already at the second closest neighboring shell of surface atoms, whilst by moving the adatom to an hcp stacking site the ferromagnetic coupling weakens without switching the sign of the magnetic coupling. In contrast to Fe, Cr-adatom shows an antiferromagnetic coupling for the nearest and the second-nearest neighbors independently from its stacking site.

Supplementary Note 2 - Simple model: interaction energy between an atom and a non-collinear dimer

Instead of an adatom deposited on a magnetic substrate, we consider here the simple case of an atom interacting with a dimer. In other words, the dimer replaces the magnetic substrate. We utilize the Heisenberg hamiltonian:

$$H = -J_{\text{ad-sub}}\left(\hat{m}_{\text{ad}} \cdot [\hat{m}^1_{\text{sub}} + \hat{m}^2_{\text{sub}}]\right) - J_{\text{sub}}\,\hat{m}^1_{\text{sub}} \cdot \hat{m}^2_{\text{sub}} - \vec{D}_{\text{sub}} \cdot \left(\hat{m}^1_{\text{sub}} \times \hat{m}^2_{\text{sub}}\right), \tag{1}$$

where J corresponds to the Heisenberg exchange interaction (HEI) either within the dimer, J_{sub} being positive, or between the adatom and one of the dimer's atoms, $J_{\text{ad-sub}}$, which can be either positive (negative) if the coupling is FM (AFM). The Dzyaloshinskii-Moriya interaction

(DMI) between the substrate atoms (here the dimer), $\vec{D}_{\text{sub}}$, is a vector defining the plane where the moments of the dimer rotate. After getting the energies minimizing the previous hamiltonian, considering, as done in the main text, the two cases: (i) adatom on top of a ferromagnetic dimer, away from the non-collinear dimer and (ii) the adatom on top of the dimer, we find that the binding energy can be approximately written as:

$$\Delta E = \Delta E_{\text{HEI}} + \Delta_{\text{DMI}}, \tag{2}$$

which if positive (negative) indicates repulsion (pinning)

$$\Delta E_{\text{DMI}} = -2\Delta E_{\text{HEI}} = \frac{D_{\text{sub}}^2}{J_{\text{sub}}}\left(\frac{2\Delta J_{\text{sub}} + |J_{\text{ad-sub}}|}{2J_{\text{sub}} + |J_{\text{ad-sub}}|}\right), \tag{3}$$

where ΔJ_{sub}, being always negative, is the change of the dimer's HEI induced by the presence of the adatom. In this simplified model, the DMI contribution counteracts the one emanating from HEI. The sign of the binding energy is dictated by the sign of ΔE_{DMI}. Interestingly, if $2|\Delta J_{\text{sub}}| > |J_{\text{ad-sub}}|$, pinning would be favored.

Supplementary Note 3 - Spin-flop state: the non-collinear Cr dimer on a ferromagnetic substrate

Considering the case of Cr dimer being antiferromagnetic and deposited on the ferromagnetic PdFe/Ir(111) substrate, where each of the adatom couple antiferromagnetically to the surface atoms can generate magnetic frustration leading to a non-collinear magnetic state. The ground state can be either non-collinear, the so-called, spin-flop state [1–4], collinear ferrimagnetic or collinear where

the dimer's moments are aligned antiparallel with respect to the surface magnetization.

We consider the following Heisenberg model, where for simplification we fix the direction of the magnetic moments of the ferromagnetic substrate and neglect DMI. Taking into account only nearest-neighbor HEI and neglecting the rotation of Fe moments of the substrate, we rewrite the Hamiltonian for the dimer in terms of the tilting angles θ_1 and θ_2 of the two Cr atoms (the azimuthal angles ϕ do not enter the expression because of symmetry reasons):

$$H = -J_{\text{ad-ad}} \cos(\theta_1 - \theta_2) - 3J_{\text{ad-sub}}(\cos\theta_1 + \cos\theta_2). \tag{4}$$

The adatom-adatom HEI, $J_{\text{ad-ad}}$, is antiferromagnetic similarly to the adatom-substrate HEI, $J_{\text{ad-sub}}$. We note the two extreme cases arising from this Heisenberg Hamiltonian: (i) $|J_{\text{ad-sub}}| \gg |J_{\text{ad-ad}}|$ leads to the stabilization of the collinear FM or AF configuration (adatom-like behavior) and (ii) $|J_{\text{ad-sub}}| \ll |J_{\text{ad-ad}}|$ leads to antiferromagnetic coupling within the dimer if $J_{\text{ad-ad}} < 0$. Within the Heisenberg model the FI solution and the non-collinear solution with $\theta = 90°$ have the same energy.

For the case of the spin-flop state found in the main manuscript, we have the angles $\theta_1 = \theta_2 = \theta/2$ defining the non-collinear (NC) solution:

$$\cos(\theta) = -\frac{3}{2}\frac{J_{\text{ad-sub}}}{J_{\text{ad-ad}}} \text{ for } J_{\text{ad-sub}} < \frac{2}{3}J_{\text{ad-ad}}. \tag{5}$$

We note that if $|J_{\text{ad-sub}}| > \frac{2}{3}|J_{\text{ad-ad}}|$, the angle is not defined and the solution has to be collinear. Considering the type of interactions characterizing the system, the moments of the two Cr adatoms

would align antiparallel to those of the substrate. If the angle is defined, the ground state is non-collinear and the energy difference with the ferrimagnetic state is given by:

$$\Delta E_{\mathrm{NC-FI}} = -\frac{9}{2}|J_{\text{ad-sub}}| \tag{6}$$

Supplementary Note 4 - Neel states of the Cr compact trimer on a ferromagnetic substrate

Here we address the two non-collinear magnetic states that can characterize the compact Cr trimer deposited on the ferromagnetic PdFe/Ir(111) surface. Because of the antiferromagnetic coupling between the Cr adatoms, their ground state is given by the Neel state with the rotation angle between the moments given by 120° if the substrate is non-magnetic. Because of the surface magnetization, the three moments can lie in the plane perpendicular to the surface magnetization but with a slight tilting giving a z-component anti-parallel to the surface moments. This defines configuration $\perp$, with the polar angle $\theta_{\perp}$ is the same for the three moments with the difference in their azimuthal angle $\Delta\phi_{\perp} = 120^{\text{o}}$, which defines configuration $\perp$. Configuration || corresponds to the case the three moments lie in the plane incorporating the surface magnetization. Here, one of the Cr moments is forced to align parallel to the surface moments, i.e. $(\theta, \phi) = (0, 0)$, in contrast to the majority of the moments of the adatoms, which align in the opposite direction but with a small tilting. The angles are given by $(\theta_{||}, 0)$ and $(\theta_{||}, 180^{\text{o}})$.

Utilizing the Heisenberg model introduced in the previous section where once more we ne-

glect here the impact of DMI, we obtain the following energies for both configurations:

$$E_{||} = -J_{\text{ad-ad}}\left(2\cos\theta_{||} + \cos 2\theta_{||}\right) - 3J_{\text{ad-sub}}\left(2\cos\theta_{||} - 1\right) \tag{7}$$

$$E_{\perp} = -\frac{3}{2}J_{\text{ad-ad}}\left(1 + \cos\theta_{\perp}\right) - 9J_{\text{ad-sub}}\cos\theta_{\perp} \tag{8}$$

with the polar angles given by

$$\cos\theta_{||} = -\frac{1}{2}\left(1 + 3\frac{J_{\text{ad-sub}}}{J_{\text{ad-ad}}}\right), \tag{9}$$

and

$$\cos\theta_{\perp} = -\frac{9}{2}\frac{J_{\text{ad-sub}}}{J_{\text{ad-ad}}} \tag{10}$$

The energy difference between the two states is given by:

$$E_{||} - E_{\perp} = -3|J_{\text{ad-ad}}| - 6|J_{\text{ad-sub}}| + \frac{45}{8}\frac{J^2_{\text{ad-sub}}}{|J_{\text{ad-ad}}|}, \tag{11}$$

where one realizes that getting one or the other configuration depends on the subtle competition of magnetic interactions. For instance, this model predicts that if $\frac{15}{8}\frac{J^2_{\text{ad-sub}}}{|J_{\text{ad-ad}}|} < (|J_{\text{ad-ad}}| + 2|J_{\text{ad-sub}}|)$ configuration $||$, i.e. the one found in the ab-initio calculations shown in the main text, should be the lowest in energy.

Supplementary References

1. Lounis, S., Mavropoulos, P., Dederichs, P. & Blügel, S. Noncollinear Korringa-Kohn-Rostoker Green function method: Application to 3d nanostructures on Ni (001). *Physical Review B* **72**, 224437 (2005).

2. Holzberger, S., Schuh, T., Blügel, S., Lounis, S. & Wulfhekel, W. Parity effect in the ground state localization of antiferromagnetic chains coupled to a ferromagnet. *Physical review letters* **110**, 157206 (2013).

3. Lounis, Samir and Mavropoulos, Phivos and Zeller, Rudolf and Dederichs, Peter H. and Blügel, Stefan. Noncollinear magnetism of Cr and Mn nanoclusters on Ni(111): Changing the magnetic configuration atom by atom. *Phys. Rev. B* **75**, 174436 (2007).

4. Lounis, S. Non-collinear magnetism induced by frustration in transition-metal nanostructures deposited on surfaces. *J. Condens. Matter Phys.* **26**, 273201 (2014).
